# Primary Immune Thrombocytopenia: A Translational Research Model for Autoimmune Diseases

**DOI:** 10.3390/jcm8111971

**Published:** 2019-11-14

**Authors:** Emmanuel Andrès

**Affiliations:** 1 Department of Internal Medicine, Diabetes and metabolic disorders, Medical Clinic B, University Hospital of Strasbourg, 67084 Strasbourg, France; emmanuel.andres@chru-strasbourg.fr; Tel.: 33-3-88-11-50-66; Fax: 33-3-88-11-62-62; 2 Referral Center of Immune Cytopenias, University Hospital of Strasbourg, 67084 Strasbourg, France; 3 Institute of Physiology, FMTS-EA 3072, Faculty of Medicine, University of Strasbourg, 11 Humann Street, 67000 Strasbourg, France

Primary immune thrombocytopenia (ITP), formally known as idiopathic thrombocytopenic purpura, is a multifactorial autoimmune disease that is both idiopathic (cause unknown) and rare [1]. Its incidence is estimated at between 1.6 and 6.6/100,000/year, depending on whether the region studied was Europe or the United States [2]. Its age-adjusted prevalence is estimated at 9.5/100,000. 

ITP is characterized by accelerated immune-mediated platelet destruction and inadequate bone marrow platelet production that leads to a platelet blood count of less than 100 × 10^9^/L [1,2]. Its clinical presentation varies from one individual to the next, ranging from asymptomatic to diverse hemorrhagic manifestations, such as petechial purpura, ecchymosis, hematoma, and internal hemorrhage [2].

The death rate from hemorrhage has been estimated at between 1% and 3% per year. Five-year mortality can be as high as 47% in patients over 60, against 2.2% in those under 40 [2,3]. 

The therapeutic management of this disorder is currently relatively well standardized, being based around expert guidelines [2,4]. Indeed, something worth noting in this regard is that it has mainly been the study of the pathophysiology of ITP that has underpinned the rationale behind the development of new treatments and has enabled the emergence of new treatment strategies. For this reason, ITP strikes us as a possible candidate for a translational research model, particularly in the area of autoimmune diseases. 

In this context, the paper published by Zufferey et al. in the *Journal of Clinical Medicine* is a key reference on the pathophysiology of ITP [5]. This is evidenced by its being one of the most frequently downloaded and cited papers of that journal. Like many other excellent papers, it has contributed to making the *Journal of Clinical Medicine* a reference text in the world of medicine, its impact factor of 5.688 classing it among the top 10 in its category (*https://www.scimagojr.com/journalsearch.php?q=21100873116&tip=sid&clean=0*).

In their paper, Zufferey et al. described current knowledge on the pathophysiology of ITP, as well as certain novel findings regarding new research into the T-cell immune response, the role of regulatory T-cells (Tregs), the role of dendritic cells, and the involvement of the spleen in the immune response [5]. Hence, these authors opened new avenues to understanding the disease pathophysiology and new possibilities for treatment. 

Here, we would like to go back over two points developed by the team of Bonnotte and Godeau, which are less well understood, but which seem to us of particular interest: the role of T-cells and the dysregulation of the immune response [6]. In this setting, the CD4+ and CD8+ T-cells are major players in the pathophysiology of ITP. 

In ITP, a disruption of the balance between helper T-cell 1 (Th1) and Th2 is observed, leading to Th1 dominance [5,6]. This is evidenced by an increase in the production of interferon-γ, as well as a decrease in the secretion of interleukin (IL-4) by T-cells [6,7]. Additionally, analysis of the hypervariable region (CDR3) of the β-chain of the T-cell receptor shows that T-cells constitute an oligoclonal population. These abnormalities are reversible in responders to rituximab [7]. At the same time, these autoreactive T-cells follow an anti-apoptotic pathway—an intracellular increase in the anti-apoptotic protein B-cell lymphoma-2 (Bcl-2), and a decrease in the proapoptotic protein Bcl-2-associated X protein—but this is also corrected by treatment [7]. Lastly, during ITP these autoreactive CD4+ T-cells contribute to the activation and affinity maturation of B-cells via CD40–CD154 interactions in splenic germinal centers [5,6]. The role of cytotoxic T-cells (Tc) has been little documented, the exception being the demonstration of increased gene expression in the perforin/granzyme system. These latter proteins are involved in cell lysis when the disease is active, suggesting that circulating CD8+ T-cells contribute to platelet destruction [8]. Analysis of intracellular cytokines in ITP patients has also revealed a Tc1/Tc2 imbalance, similar to that observed with Th-cells [7]. Lastly, the increased recruitment of these T-cells into bone marrow leads to the destruction of the progenitors of platelets, the megakaryocytes, thereby contributing to insufficient platelet production [8].

Autoreactive T-cells recognize the platelet glycoprotein complex GPIIb/IIIa [5]. Thus, their presence in both healthy subjects and ITP patients [5,6] hints at a breakdown in peripheral tolerance in this disease. This breakdown in tolerance may be due to a quantitative and/or qualitative deficit of Tregs. Originating in the thymus, this CD4+ T-cell subpopulation constitutively and intensely expresses CD25, the α-chain of the IL-2 receptor [5,9], as well as the transcription factor forkhead box P3, which plays a primordial role in their genesis as well as in maintaining their peripheral immunosuppressive function. Whether a quantitative deficit of Tregs exists in ITP continues to be debated. Some authors have observed a quantitative deficit in patients compared with control subjects [5,7,9]. This abnormal finding is reversed by corticosteroid therapy or rituximab [5]. However, others have noted similar levels in ITP patients and controls [5,9]. That said, all authors report a functional deficit of Tregs in ITP which results in a reduction in their ability to inhibit the proliferation of effector T-cells [5,7,9].

These pathophysiological insights may lead to new treatment approaches in the future, making it possible to modify the natural history of the disease, or even beat it definitively.
